# Internal exposure to perfluoroalkyl substances (PFASs) and biological markers in 101 healthy 1-year-old children: associations between levels of perfluorooctanoic acid (PFOA) and vaccine response

**DOI:** 10.1007/s00204-020-02715-4

**Published:** 2020-03-29

**Authors:** Klaus Abraham, Hans Mielke, Hermann Fromme, Wolfgang Völkel, Juliane Menzel, Matthias Peiser, Fred Zepp, Stefan N. Willich, Cornelia Weikert

**Affiliations:** 1 Department Food Safety, German Federal Institute for Risk Assessment (BfR), Max-Dohrn-Str. 8-10, 10589 Berlin, Germany; 2 Department Exposure, German Federal Institute for Risk Assessment, Berlin, Germany; 3 Department Pesticide Safety, German Federal Institute for Risk Assessment, Berlin, Germany; 4 Institute and Clinic for Occupational, Social and Environmental Medicine, University Hospital, LMU Munich, Munich, Germany; 5 Department of Chemical Safety and Toxicology, Bavarian Health and Food Safety Authority, Munich, Germany; 6 Children’s Hospital, University Medical Center, Mainz, Germany; 7 Institute for Social Medicine, Epidemiology and Health Economics, Charité – Universitätsmedizin Berlin, Berlin, Germany

**Keywords:** Perfluoroalkyl substances, Vaccine antibodies, Immune response, Cholesterol, Children

## Abstract

Perfluoroalkyl substances (PFASs) are a complex group of man-made chemicals with high stability and mobility leading to ubiquitous environmental contamination and accumulation in the food chain. In human serum/plasma samples, perfluorooctanoic acid (PFOA) and perfluorooctane sulfonate (PFOS) are the lead compounds. They are immunotoxic in experimental animals, and epidemiological studies provided evidence of a diminished production of vaccine antibodies in young children. However, information on children of the first year of age is missing but relevant, as they have a relatively high exposure if breastfed, and may have a higher susceptibility as their immune system is developing. In a cross-sectional study with 101 healthy 1-year-old children, internal levels of persistent organic pollutants and a broad panel of biological parameters were investigated at the end of the 1990s. Additional analysis of PFASs resulted in plasma levels (mean ± SD) of PFOA and PFOS of 3.8 ± 1.1 and 6.8 ± 3.4 µg/L, respectively, in the 21 formula-fed children, and of 16.8 ± 6.6 and 15.2 ± 6.9 µg/L in the 80 children exclusively breastfed for at least 4 months. The study revealed significant associations between levels of PFOA, but not of PFOS, and adjusted levels of vaccine antibodies against *Haemophilus influenza* type b (Hib, *r* = 0.32), tetanus (*r* = 0.25) and diphtheria (*r* = 0.23), with no observed adverse effect concentrations (NOAECs) determined by fitting a ‘knee’ function of 12.2, 16.9 and 16.2 µg/L, respectively. The effect size (means for PFOA quintiles Q1 vs. Q5) was quantified to be − 86, − 54 and − 53%, respectively. Furthermore, levels of PFOA were inversely associated with the interferon gamma (IFNɣ) production of ex-vivo lymphocytes after stimulation with tetanus and diphtheria toxoid, with an effect size of − 64 and − 59% (means Q1 vs. Q5), respectively. The study revealed no influence of PFOA and PFOS on infections during the first year of life and on levels of cholesterol. Our results confirmed the negative associations of PFAS levels and parameters of immune response observed in other epidemiological studies, with high consistency as well as comparable NOAECs and effects sizes for the three vaccine antibodies investigated, but for PFOA only. Due to reduction of background levels of PFASs during the last 20 years, children in Germany nowadays breastfed for a long duration are for the most part not expected to reach PFOA levels at the end of the breastfeeding period above the NOAECs determined.

**Electronic supplementary material:**

The online version of this article (10.1007/s00204-020-02715-4) contains supplementary material, which is available to authorized users.

## Introduction

Perfluoroalkyl substances (PFASs) are a complex group of man-made chemicals with a chemical structure consisting of a perfluorinated carbon tail (hydrophobic/lipophobic end) and an anionic head group (hydrophilic end). Beginning in the middle of the last century, they are used for several industrial applications including the production of water repellent clothes or cookware. The compounds have a high stability and mobility, and after release from industrial sources and everyday objects, these properties have led to ubiquitous environmental contamination. PFASs in part accumulate in the food chain and are found at relatively high levels in human blood samples, as the half-life of several years in man is—compared to other species—exceptionally long. During the last decades, the C8-compounds perfluorooctanoic acid (PFOA) and perfluorooctane sulfonate (PFOS) were found as the lead compounds with levels in the one- to two-digit microgram per liter (µg/L) range representing background exposure (Vestergren and Cousins 2009; Sunderland et al. 2019; EFSA 2018).

Besides other effects, PFOA and PFOS were found to cause immune suppression in laboratory animals, with strongest evidence for the suppression of the T cell dependent antibody response. Results vary depending on the species/strain, sex, and route of exposure, and the immunotoxic mode of action in animals has not yet been elucidated (Chang et al. 2016; NTP 2016; ATSDR 2018; EFSA 2018). Results of epidemiological studies suggest that moderate to high background exposure to PFOA and PFOS also adversely affect serum antibody response following vaccination in children. This has been mainly proposed by studies from the Faroe Island, conducted in up to 587 children. These studies in part revealed inverse associations between current levels of PFOA and PFOS and levels of the vaccine antibodies against tetanus and diphtheria at the age of 5 years as well as at the age of 7 and 13 years after booster vaccinations at 5 years of age (Grandjean et al. 2012, 2017a, b). However, only a part of the inverse associations reached significance level, and the consistency regarding the immunotoxic potencies of PFOA and PFOS, the time course of diminished antibody levels as well as the dependency on the infectious agent (against which the vaccination was carried out) is limited in these studies. Furthermore, in part significant inverse associations were observed between maternal levels of PFOA and PFOS at birth and levels of vaccine antibodies at the age of 3 or 5 years (Granum et al. 2013; Grandjean et al. 2012, 2017a, b). Weaker or no associations were observed in adolescents and adults (Grandjean 2017b, Looker et al. 2014; Stein el al. 2016a, b), suggesting a lower susceptibility of these age groups compared to young children. Currently, there is an ongoing scientific debate regarding the assessment of the evidence available and the clinical adversity/relevance of the results of these epidemiological studies (DeWitt et al. 2019). Regarding risk assessment, the CONTAM Panel of the European Food Safety Authority (EFSA) was the first international scientific body using the data of epidemiological studies (critical effects: increase of cholesterol and decrease of antibody response after vaccination) for the derivation of health based guidance values for PFOA and PFOS in 2018 (EFSA 2018).

Data on vaccine antibodies in association with levels of PFASs are only available for children 3 years of age and older, but are currently missing for younger children. However, the latter group is even higher exposed if breastfed for a long duration, reaching a peak internal exposure to PFASs at the end of the breastfeeding period; thereafter, internal exposure decreases due to much lower external exposure after weaning and due to dilution by growth of body mass (Verner et al. 2016). Furthermore, children in the first year of life are suggested to have a higher susceptibility, as their immune system is developing. They receive basic immunization within the first months of life and a reduced immune response would in principal be undesirable.

In this article, we report on the re-evaluation of a study in 101 healthy 1-year-old children performed at the end of the 1990s (Abraham 2000) after the additional analysis of PFASs in stored plasma samples in 2019. The original focus of the study was on possible effects of polychlorinated dibenzo-*p*-dioxins and dibenzofurans (PCDD/Fs, ‘dioxins’) and biphenyls (PCBs) on biological markers measured in blood, with special emphasis on the immune system. Children predominantly were breastfed for a long duration and therefore had relatively high levels of internal exposure to persistent organic pollutants (POPs). At the levels of background exposure at the end of the 1990s, results of the study suggested a missing impact of dioxins, PCBs and some ‘old’ pesticides on the biological markers investigated (Abraham 2000). As a targeted approach, the re-evaluation focusses on the possible effects of PFASs reported in other epidemiological studies: vaccine response and immune system, fat metabolism as well as thyroid status.

## Methods

### Study concept

Children exclusively breastfed for several months reach relatively high levels of persistent organic pollutants at the end of the breastfeeding period—at a time of possible relatively high susceptibility for effects on the immune system (WHO/IPCS 2012). A broad spectrum of biological parameters should be measured in blood at the end of the first year of life together with the internal exposure of relevant contaminants (thought to be at the end of the 1990s: dioxins and PCBs as well as important ‘old’ pesticides and heavy metals). To get a high variation of the levels of children’s internal exposure, they should be either formula-fed or breastfed for a long duration. To further increase the variation, breastfed children should also be recruited from dioxin hotspot regions in addition to those from Berlin. Furthermore, blood should also be collected from the mother to get information on the accumulation of the contaminants during breastfeeding.

### Study design

To reduce the influence of possible confounders, strong in- and exclusion criteria were applied: children of German parents had to have an age within the 4-weak-span of 341 to 369 days, and had to be either breastfed for maximally 2 weeks (‘formula-fed’ children) or breastfed equivalent to a duration of exclusive breastfeeding of 4 months or longer. They had to be healthy during their previous life (normal development and weight gain, no former preterm or small for gestational age infants, no chronic diseases including atopic eczema, no medication apart from vitamin D and fluoride). They had to be free of acute infections (no fever and good general health condition within the last 2 weeks), but slight sneezing or coughing was accepted. Children had to be vaccinated according to the recommended vaccination schedule, with at least two vaccinations against diphtheria and tetanus. Furthermore, it had to be possible to transport the blood to the lab within 4 h. The study protocol was approved by the ethics committee of the Virchow-Klinikum of the Free University Berlin, one of the predecessors of the Charité – Universitätsmedizin Berlin, in November 1995.

Recruitment was performed via announcement in newspaper and via pediatricians. All parents gave their informed consent in writing prior to their inclusion in the study. Between June 1997 and May 1999, a total of 101 children were examined. A further 32 mother–child pairs were initially recruited, but the examination could not take place for various reasons: 16 mothers withdrew their consent for various reasons (e.g. concerns about blood withdrawal), ten children could not be examined due to infections existing in the time window (no further postponement possible), and three children had received no or only one vaccination against diphtheria and tetanus. In three children, the blood collection was not successful.

### Investigations of the children and their mothers

The examinations took place at the children’s home at the time the child usually awakes in the morning. To reduce pain, an Emla® plaster (Astra, Wedel, Germany) containing local anesthetics was placed on the back of both hands. After the physical examination including measurement of height and body weight, blood (15 mL heparin blood, 0.5 mL ethylenediamine tetraacetic acid (EDTA) blood) was collected via an open cannula inserted into a vein on the back of the hand. To keep the child as calm as possible during fixing the hand, breastfeeding or drinking of the morning milk bottle was encouraged. Thereafter, blood (40 mL heparin blood, 2 mL EDTA blood) was collected from the mother. An extensive interview was conducted regarding important parameters of the previous life of mother and child, including detailed data on child feeding (breastfeeding, introduction of complementary foods, weaning), previous infections (number, kind, antibiotic treatments), vaccinations and social status. In 36 children, a second blood sampling (approximately 7 mL) took place on average 30 days after the first one, mainly to check for conspicuous laboratory results of the first blood sampling. The most frequent reason was an increase in plasma alkaline phosphatase enzyme activity (higher than 700 U/L), which was observed in 14 children upon the initial blood sampling. The second investigation was used to repeat as much as possible of the entire study program (except for POP and heavy metal analyses), so that the intra-individual variability could be determined for the biological parameters investigated within a few weeks. All investigations were done by the same pediatrician (K.A., at that time employee of the Charité, Department of Pediatrics, Division of Pneumonology and Immunology, Director: Prof. Dr. Ulrich Wahn).

### Laboratory measurements

In the following, a brief description of the laboratory methods applied is given. A more detailed description is given in the 90-page report (Abraham 2000). In general, strong criteria were applied regarding method validity and stability over time (a few parameters missing this criterion were excluded from the evaluation).

Blood count was determined from the EDTA blood using an automatic cell counter (Cell Dyn 3500, Abbott). After separation of the heparin blood for assessment of lymphocyte subpopulations (1.0 mL), cell proliferation using whole blood (1.5 mL) and analyses of heavy metals (1.3 mL, frozen at − 80 °C), the remaining blood was centrifuged (15 min, 700 g).

The following plasma measurements were made at the day of blood draw on a Modular PP (Roche) clinical chemistry analyzer using routine methods: C-reactive protein (CRP; immunoturbidimetry/Wako), total protein, albumin (bromocresol green/Roche), cholesterol, triglycerides, total bilirubin, alanine aminotransferase (ALT), γ-glutamyl transpeptidase (GGT), lactate dehydrogenase (LDH), iron (ferrozine/Roche), ferritin (latex-enhanced immunoturbidimetry/Roche), transferrin and immunoglobulins A, G, and M (all immunoturbidimetry/Dako). Furthermore, plasma (1.9 mL) was frozen in aliquots and stored at − 80 °C until further analysis. At the end of the sample collecting period, the following measurements were done in a batch. CRP was determined a second time using a high-sensitivity adaptation of a latex-enhanced immunoturbidimetric assay kit (Quantex CRP, Biokit, detection limit 0.2 mg/L) to a Cobas Fara II centrifugal analyzer (Roche). Furthermore, thyroid function parameters (total triiodothyronine, T3; total thyroxine, T4; free thyroxine, fT4; thyroid-stimulating hormone, TSH; thyroxine-binding globulin, TBG) were measured on an Autodelfia analyzer (immunofluorometry/Wallac). IgG subclasses (The Binding Site) were determined using a turbidimetric measurement, and IgE using an ELISA technique (Pharmacia). Specific vaccine antibodies were measured using ELISA kits according to the instructions of the company (The Binding Site) for tetanus toxoid IgG (MK010, unit IU/mL, 1 IU/mL = 17 mg/L) and IgG1 antibodies (MK011, unit mg/L) as well as for *Haemophilus influence* type b (Hib) polysaccharide capsule IgG antibodies (MK016, unit mg/L). All these measurements were run in duplicate. Furthermore, retinoids were analysed for questions regarding the influence of minimal inflammation (Abraham et al. 2003), but are not considered for this evaluation.

The remaining plasma (3–5 mL) was also frozen at − 80 °C until analysis of persistent organochlorine compounds at the former ERGO Forschungsgesellschaft (Hamburg, Germany): 2378-substituted PCDDs and PCDFs, non dioxin-like PCBs (ndl-PCBs, No. 28, 52, 101, 138, 153, 180), mono-ortho-PCBs (No. 105, 118, 156), coplanar PCBs (No. 126, 169), as well as the ‘old’ organochlorine pesticides dieldrin, dichlorodiphenyltrichloroethane (DDT) and its metabolites (op-DDD, pp-DDD, op-DDT, pp-DDT, op-DDE, pp-DDE), hexachlorobenzene (HCB) and ß-hexachlorocyclohexane (ß-HCH). Dioxin analysis was performed using an optimized isotope dilution method with quantification on HRGC/HRMS. Details are described elsewhere (Päpke 1997; Abraham 2000). Results were based on total plasma fat calculated from cholesterol and triglycerides values according to Phillips et al. (1989). For the formula-fed infants, individual analyses were performed in five children and their mothers only; for the 16 other children, two pool samples were analysed to reduce the number of compounds below the limit of detection (about 1 pg per g fat in case of 2378-TCDD for an individual analysis). For consideration as possible confounders, I-TEq were calculated from levels of PCDDs and PCDFs using the toxicity equivalency factors (TEFs) of Nato/CCMS, as well as WHO-TEq from levels of PCDD/Fs and those of coplanar and mono-ortho PCBs using the TEFs published 1998 (Van den Berg et al. 1998). Quantifiable ndl-PCBs were considered as sum of PCBs No. 138, 153 and 180.

Furthermore, determination of heavy metals in whole blood of child and mother (lead, cadmium, mercury) was performed at the German Federal Environment Agency using atomic absorption spectrometry as described in Benemann et al. (2004).

Lymphocyte proliferation after stimulation was investigated using a whole blood method: 1 + 7 dilution with serum-free culture medium (AIM V, Thermo Fisher Scientific) on a 96-well plate with 100 μL blood dilution plus 100 μL stimulant in the medium per well, culture (37 °C, 5% CO_2_, 100% humidity) over 3 days with phytohemagglutinin (PHA), conconavalin A (ConA), poke weed mitogen (PWM), and over 5 days with tetanus and diphtheria toxoid, Staphylococcal enterotoxin B (SEB) and Candida antigen (each triplets with three different concentrations and twelve wells medium-control), 6 h pulse (1 μCi per well) with ^3^H-thymidine (Amersham, 5 Ci/mmol) at the end of culture time and freezing of plates at − 18 °C, ‘harvesting’ with an automatic cell harvester within few days, measurement in a ß-counter after transfer of the filter plates in tubes and addition of 2 mL scintillation cocktail. Evaluation was based on the maximum proliferation rate (mean of triplet) observed for the three concentrations of the stimulant.

Lymphocyte subpopulations were analysed after three-color staining (PerCP, PE and FITC) with fluorescence-marked monoclonal antibodies (immunophenotyping): 25 μL whole blood each incubated with 18 combinations (Abraham 2000) for 15 min at room temperature, lysis of the erythrocytes (Ortho Diagnostics Systems, 10 min), wash of the cells and measurement of 50,000 leukocytes per combination by a flow cytometer FACS Calibur (Becton Dickinson).

After centrifuging of the whole blood and removal of the plasma, the residual material (buffy coat and erythrocytes) was diluted with culture medium. Peripheral blood mononuclear cells (PBMCs) were subsequently isolated via Ficoll gradient (15 min, 700 g) and washed. Cells (3 × 10^6^ per mL culture medium with a concentration of 10 µg PHA per mL or medium only) were incubated (37 °C, 5% CO_2_, 100% humidity) to recover the culture supernatants after 48 h. Likewise, culture supernatants were removed after 72 h of the whole blood incubations with tetanus and diphtheria toxoid (see above; carried out beginning with participant No. 47, therefore *n* = 55 only). The material was frozen at − 80 °C for later measurement of the cytokines interleukin 5 and 10 (IL-5, IL-10; in case of stimulation with PHA only) as well as interferon gamma (IFNɣ) using ELISA kits (Pharmingen).

After Ficoll separation, the remaining material (granulocytes and erythrocytes) was washed, and granulocytes were subsequently isolated via a Percoll gradient (densities 1.076 and 1.095, 15 min, 700 g). The contaminating erythrocytes were lysed, the cells washed and adjusted to a number of 5 × 10^6^ per mL. The phagocytosis and burst activity of the granulocytes was determined by measuring chemiluminescence over 40 min in a luminometer (with the bacterial wall component zymosan previously opsonized by pooled or individual plasma).

### Analyses in 2019

After release of the EFSA opinion on PFOA and PFOS in December 2018, we decided to conduct further measurements using two remaining plasma aliquots (125 µL each, continuously frozen at − 80 °C). This study extension was approved by the ethics committee of the Charité (EA2/090/19).

PFASs were measured at the Bavarian Health and Food Safety Authority. The following compounds were analysed using 100 µL plasma: PFOA, PFOS, perfluorobutane sulfonate (PFBS), perfluorohexane sulfonate (PFHxS), perfluorohexanoic acid (PFHxA), perfluorononanoate (PFNA), perfluorodecanoate (PFDA), perfluorododecanoate (PFDoDA), and 3H-perfluoro-3-[(3-methoxy-propoxy)propanoate] (ADONA). Sample preparation, analysis and quality criteria have been previously described in detail by Mosch et al. (2010). Briefly, the perfluorinated substances and the corresponding isotope-labeled internal standards were purchased from Wellington Laboratories (Ontario, Canada). For analysis, an online extraction LC–MS/MS system was used, and the compounds were quantified with a triple-stage quadrupole mass spectrometer (API 5500 QTRAP™ Applied Biosystems, Darmstadt, Germany) equipped with a TurboIonSpray® interface. Limit of quantification (LOQ) in plasma samples based on a tenfold peak-to-noise ratio was 0.25 µg/L. Values below this limit were considered with half the LOQ.

As the strongest effect on vaccine antibodies on the Faroe Islands was observed for diphtheria (Grandjean et al. 2012), the antibodies against diphtheria toxoid were additionally measured in duplicate using ELISA kits according to the instructions of the company (MK014, The Binding Site Group Ltd, Birmingham, UK).

### Statistical evaluation

The main focus of this targeted evaluation was on possible association of PFASs and parameters of immune response to vaccinations against Hib, tetanus and diphtheria. The programs used were SAS (version 9.3), SPSS (version 21) and R (version 3.6.1, R Core Team 2019) with package ‘segmented’ (Muggeo 2017). All correlations reported are Spearman correlation coefficients (*r*). A threshold of *r* = 0.2 was used for spotting potentially relevant associations which roughly corresponds to *p* = 0.05 for the given cohort size of 101 children.

A linear model was fitted for each of the plasma antibody concentrations, taking into account the time since last vaccination and, if significant, the number of vaccinations. Using this model, the antibody response was then expressed using the regressed values at the time of vaccination. Further analyses were performed on these data which are referred to as *adjusted values*. The possible influence of other contaminants was analysed using the stepwise inclusion–exclusion process based on AIC (R function ‘stepAIC’), starting with the full model explaining the respective adjusted antibody concentration.

To explore a dose–response relation between levels of contaminants and antibodies, several methods were applied. First, a linear model was fitted to confirm possible associations. Then a LOESS smoother was applied as it was judged that such moving average might reveal a trend in the data. Furthermore, the data were divided in PFAS quintiles and deciles. Plots were drawn as box-plots and as plots contrasting the empirical distribution to the normal distribution of the PFOA quintiles. If an ANOVA showed differences between the quintile/decile groups, a no observed adverse effect concentration (NOAEC) was derived as the highest-dose quantile below the first one showing a significant difference to the lowest-dose quantile. Furthermore, the breakpoint of a piecewise linear regression with two segments and left slope null (‘knee’ function) was estimated (least squares estimate by R package ‘segmented’).

## Results

### Study population

Of the 101 children (51 boys, 50 girls) examined, 21 were formula-fed, and 80 were breastfed for at least 4 months, including 27 children from a dioxin hotspot outside Berlin (former copper smelter without filter system). None of the children were subsequently excluded from the study. The median age of the children was 351 days (50.1 weeks, range 48.7–52.7 weeks). Basic characteristics and body measurements for the formula-fed and breastfed children are summarized in Table 1. The distribution of body measurements in the study corresponded to expectations for healthy German children, with all anthropometric data between the 3rd and 97th percentiles, except for one breastfed girl with a body weight of 7.30 kg at the age of 11.4 months slightly below the 3rd percentile.

**Table 1:** Basic data of the study population consisting of 101 healthy children

	Formula-fed infants	Breastfed infants
*N*	21	80
Sex	10 boys, 11 girls	41 boys, 39 girls
Age of mother (years)	30.3 ± 5.6	31.7 ± 3.8
Body weight of mother (kg)	64.1 ± 13.8	62.4 ± 8.9
Gestational age (weeks)	40.7 ± 1.0*	40.2 ± 1.0*
Birth weight (kg)	3.51 ± 0.39	3.52 ± 0.39
Birth length (cm)	51.7 ± 2.3	51.9 ± 2.1
Age (weeks)	50.1 ± 0.9	50.4 ± 1.1
Body weight (kg)	9.71 ± 0.97	9.36 ± 0.78
Length (cm)	75.4 ± 2.8	75.1 ± 2.3

*Statistically different (*t*-test, *p* < 0.05)

### Feeding

The 21 ‘formula-fed’ infants were never breastfed (*n* = 11) or breastfed for maximally 2 weeks (*n* = 10). The 80 breastfed children had a mean duration of exclusive breastfeeding of 5.5 months. Thirty of them were exclusively breastfed for 6 months, and eight for longer than 6 months (max 10.8 months). Twenty-nine children were still partially breastfed at the time of the examination. As an estimate for the total transfer of milk and contaminants from mother to child, the equivalent duration of exclusive breastfeeding was calculated from the information at which time each meal of mother’s milk was replaced by complementary foods (e.g. 2 months with three breastfeeding meals and three other meals were calculated as 1 month of exclusive breastfeeding). This value corresponds to a duration of breastfeeding with abrupt weaning and was on average 7.4 months (= median, range 3.6–11.1 months). Thirty-six mothers had already breastfed at least one other child, on average with a total equivalent duration of exclusive breastfeeding of 7.3 months for all siblings (range 1.8–18.5 months). Compared to the children from Berlin, the 27 children from a dioxin hotspot outside Berlin were breastfed significantly shorter in terms of the equivalent duration of exclusive breastfeeding (mean 6.6 vs. 7.8 months in breastfed children from Berlin) and the time of weaning. Anthropometric data of the children were not significantly different in the two regions.

### Signs of infection and sensitive CRP

All children were healthy (i.e. good general health condition, no fever, no illness specific symptoms) apart from minimal signs of airway infections (e.g. sneezing or coughing not more than twice within one hour, slight nasal obstruction without runny nose) observed in 42 children. As expected, the number of previous infections (up to 10) was mainly influenced by the number of siblings in toddler age (median: three airway infections without and six with one or more siblings). Only a relatively small number of 12 children already attended a kindergarten or a childminder. There were no significant group differences between breastfed and formula-fed children with regard to the infections they had suffered until then.

The CRP values determined by routine methods for the identification of children with inflammatory processes were all below the usually stated limit of 6 mg/L. Using a more sensitive method, sCRP revealed values below the detection limit of 0.2 mg/L in 33 children and a median of all children of 0.26 mg/L. Twenty-two children were above the triple value of the median of 0.75 mg/L (maximum 8 mg/L). Surprisingly, even at this low level, a partly highly significant association of sCRP values with parameters of the acute phase reaction was found: lower prealbumin, retinol, iron, and free thyroxine, higher thyroxine-binding protein (Abraham et al. 2003). The reason for higher sCRP values is presumably the activation of granulocytes caused by bacterial superinfections often developing in small children with viral airway infections. This was not apparent from the clinical investigation: 33% of children with and 14% of those without mild signs of infection had sCRP values above 0.75 mg/L.

### Reproducibility of results for biological parameters

The second blood sampling carried out in 36 children on average 30 days after the first one allowed repeating measurements of laboratory parameters to check their biological reproducibility over time. This information was rated relevant as it makes sense to consider an impact of a persistent contaminant on a biological parameter only if the parameter has a low intra-individual variability within weeks, as compared to the inter-individual variability. As a measure of reproducibility, the average absolute difference between the two measurements was divided by the standard deviation (SD) of the parameter calculated from the measurement in all children. These values are compiled for all parameters in the project report (Abraham 2000). For the parameters identified in the following as possibly influenced by the levels of PFASs, low intra-individual variability was observed for specific antibody levels (between 19 and 26% of SD, due to huge inter-individual variability) as well as for the lymphocyte subpopulation CD27- as percentage of CD8 cells (22% of SD). A higher intra-individual variability was found for CD45RO^+^CD45RA^−^ as percentage of CD8 cells (60% of SD). The value for the IL-10 production of ex-vivo lymphocytes after PHA stimulation was one of the highest (89% of SD).

### Levels of contaminants

Levels of the contaminants measured are given in Table 2 for the 21 formula-fed and the 80 breastfed children. PFOA and PFOS were quantifiable in all the 101 children, whereas in case of PFHxS and PFNA, one and 28 samples were below the LOQ, respectively. The other five PFASs (PFBS, PFHxA, PFDA, PFDoDA and ADONA) were found to be exclusively or predominantly below the LOQ. Of the lipophilic contaminants already measured at the end of the 1990s, 22 compounds were quantifiable—with a few exceptions—in all breastfed children: twelve 2378-substituted PCDD/Fs (calculated as I-TEq value), four coplanar/mono-ortho PCBs (No. 126, 169, 118, 156, considered together with PCDD/Fs as WHO-TEq (1998)), three ndl-PCBs (No. 138, 153, 180, considered as sum) as well as ß-HCH, HCB and pp-DDE. Furthermore, the levels of mercury, cadmium and lead are compiled in Table 2.

**Table 2:** Levels of persistent organic pollutants and heavy metals measured in the formula-fed and breastfed children at the age of 1 year

	Formula-fed children (*n* = 21)		Breastfed children (*n* = 80)	
	Mean ± SD	Range	Mean ± SD	Range
PFOA (µg/L plasma)	3.8 ± 1.1	1.6–6.4	16.8 ± 6.6	2.6–36.7
PFOS (µg/L plasma)	6.8 ± 3.4	2.8–19.3	15.2 ± 6.9	1.9–34.8
PFHxS (µg/L plasma)	1.7 ± 1.1	< 0.25–3.8	2.1 ± 1.3	0.3–7.1
PFNA (µg/L plasma)	0.2 ± 0.1	< 0.25–0.6	0.6 ± 0.2	< 0.25–1.4
I-TEq (pg/g fat)	2.4^a^	n.a	27.6 ± 15.1	1.7–107.0
WHO-TEq (1998) (pg/g fat)	n.c	n.a	59.7 ± 28.6	5.8–173.9
ndl-PCBs (ng/g fat)	25.2^a^	n.a	449 ± 295	43–1489
pp-DDE (ng/g fat)	38.9^a^	n.a	674 ± 501	50–2536
HCB (ng/g fat)	< 20^a^	n.a	189 ± 154	< 20–826
β-HCH (ng/g fat)	10.9^a^	n.a	60.1 ± 34.6	10.0–197.4
Hg (µg/L blood)	0.16 ± 0.12	0.1–0.5	0.23 ± 0.31	0.1–2.2
Cd (µg/L blood)	0.12 ± 0.07	0.04–0.39	0.13 ± 0.09	0.02–0.72
Pb (µg/L blood)	25.4 ± 13.0	9–57	36.1 ± 25.2	12–196^b^

*n.c.* not calculated (most levels of PCBs below LOD), *n.a.* not available (pool analyses in part)

^a^Calculated from 5 individual and 2 pool analyses

^b^Highest level in part due to drinking juices from a metal cup; second highest value: 94 µg/L blood

**Fig. 1:**
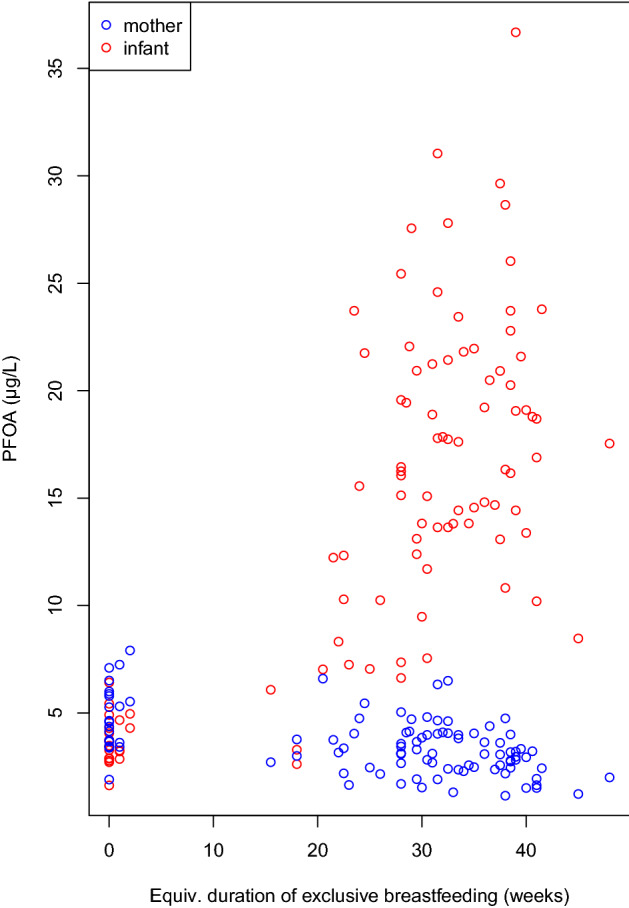
Scatter plot of plasma levels of PFOA in children and their mothers in relation to the equivalent duration of exclusive breastfeeding (*n* = 101)

The correlation of PFASs with the equivalent duration of exclusive breastfeeding was highest for PFOA (*r* = 0.68), as displayed in Fig. 1, followed by PFNA (*r* = 0.63), PFOS (*r* = 0.59), and PFHxS (*r* = 0.32). The high correlation coefficients were comparable to those of the lipophilic contaminants ndl-PCBs (*r* = 0.72), ß-HCH (*r* = 0.70), pp-DDE (*r* = 0.64), and I-TEq (*r* = 0.62). The coefficients for the correlations of the different contaminants among each other are given in the supplemental Table S1. For PFOA, highest correlation coefficients were observed with ndl-PCBs (*r* = 0.72), PFNA (*r* = 0.72), I-TEq (*r* = 0.67) and PFOS (0.67).

Depending on the degree of accumulation, an inverse association between the maternal levels about 1 year after delivery and the equivalent duration of exclusive breastfeeding was found for PFOA (*r* = − 0.48), as also displayed in Fig. 1, followed by PFNA (*r* = − 0.32), PFHxS (*r* = − 0.28), and PFOS (*r* = − 0.18). Therefore, the levels of the mothers who did not breastfed the child (*n* = 21) are expected to be the best measure for the background levels of PFASs at the end of the 1990s in Germany, and were 4.9 ± 1.5 µg/L for PFOA, 17.2 ± 6.1 µg/L for PFOS, 1.8 ± 0.9 µg/L for PFHxS, and 0.4 ± 0.2 µg/L for PFNA (mean ± SD). Detailed data on the different POP kinetics will be published in a separate paper.

### Vaccine antibodies

Evaluation of the vaccine antibodies anti-Hib (IgG), anti-tetanus (IgG and IgG1) and anti-diphtheria (IgG) in the 1-year-old children was restricted to those vaccinated two or three times against Hib (*n* = 98) and tetanus/diphtheria (*n* = 100). Tetanus IgG antibodies were measured as IU/mL (mean 0.97 IU/mL, median 0.50 IU/mL, range 0.03–18.4 IU/mL). Tetanus IgG and IgG1 antibodies strongly correlated (*r* = 0.992); therefore, the following evaluations were made for tetanus IgG1 antibodies only. Basic data of Hib, tetanus IgG1 and diphtheria antibodies are compiled in Table 3. The time since the last vaccination was 23 ± 7 weeks (mean ± SD, range 2–36 weeks) for all these vaccinations.

**Table 3:** Basic data and results of the evaluation of PFOA/PFOS influence on vaccine antibodies against Hib, tetanus and diphtheria in children vaccinated at least two times, as well as IFNɣ production of ex-vivo lymphocytes after stimulation with tetanus and diphtheria toxoid

	Hib (IgG)	Tetanus (IgG1)	Diphtheria (IgG)
Number of vaccinations	2 (*n* = 30), 3 (*n* = 68)	2 (*n* = 16), 3 (*n* = 84)	2 (*n* = 16), 3 (*n* = 84)
**Antibody levels**			
Mean/median (as measured)	9.58/2.00 mg/L	11.8/6.13 mg/L	0.512/0.259 IU/mL
Range	0.026–100 mg/L	0.286–191 mg/L	0.009–4.45 IU/mL
Correlation of adjusted levels			
With PFOA (*r*)	− 0.32	− 0.25	− 0.23
With PFOS (*r*)	− 0.05	− 0.07	− 0.02
PFOA fit knee function			
NOAEC as ‘knee’ at	12.2 µg/L	16.9 µg/L	16.2 µg/L
Adj. antibody mean ± SD (log scale)^a^	1.850 ± 0.697	1.045 ± 0.374	0.544 ± 0.345
Adj. antibody − SD **mean** + SD (lin. scale)^a^	14.2 **70.8** 353	4.68 **11.1** 26.2	1.58 **3.50** 7.72
Adj. antibody level minus − 1 SD at (Fig. 3)	27.9 µg/L	29.7 µg/L	24.7 µg/L
PFOA quantiles			
Quintiles: NOAEC at^b^	Q4 mean: 19.4 µg/L	Q4 mean: 18.9 µg/L	Q4 mean: 18.9 µg/L
Deciles: NOAEC at^b^	Q8 mean: 20.5 µg/L	Q9 mean: 22.4 µg/L	Q9 mean: 22.4 µg/L
Effect size quintiles (adj. antibody level, on lin. scale), mean Q1 vs. Q5: lower by	− 86%	− 54%	− 53%
**IFNɣ production after stimulation with the toxoid (*n* = 55)**			
Mean/median	**–**	136/67 pg(mL	26/8 pg/mL
Range	–	4–617 pg/mL	6–197 pg/mL
Effect size using PFOA tertiles, mean Q1 vs. Q3: lower by	–	− 64%	− 59%
Correlation with PFOA (*r*), *n* = 55	–	− 0.33	− 0.24
Correlation with PFOS (*r*), *n* = 55	–	− 0.10	− 0.21

^a^Antibody levels of children with PFOA below the ‘knee’ level

^b^Mean PFAS level of the highest quantile below the first one showing a significant difference to the quantile Q1

**Fig. 2:**
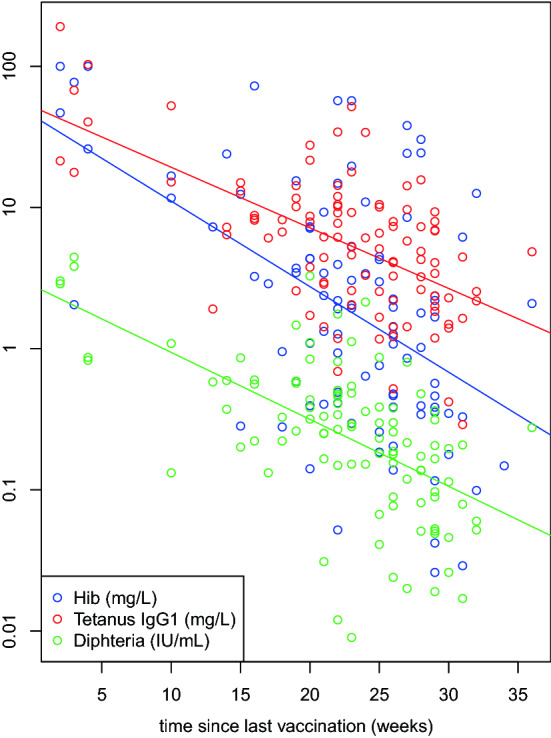
Scatter plot of levels of vaccine antibodies as measured in plasma for Hib (*n* = 98), tetanus IgG1 (*n* = 100) and diphtheria (*n* = 100), in relation to the time since the last vaccination in children with at least two vaccinations

Levels of vaccine antibodies were strongly right skewed and were therefore log-transformed (base 10) for further statistical analyses. Linear regression analysis revealed a strong influence of the time since last vaccination (Fig. 2, roughly indicating half-lives between 5 and 7 weeks on average for the three antibody levels), and a much smaller but significant influence of the number of vaccinations in case of tetanus only. For the subsequent evaluations, the antibody levels were adjusted for the time since last vaccination and additionally for the number of vaccinations in case of tetanus only.

Correlation between adjusted antibody levels and PFOA levels revealed significant associations for Hib (*r* = − 0.32, *p* = 0.001), tetanus IgG1 (*r* = − 0.25, *p* = 0.01) and diphtheria (*r* = − 0.23, *p* = 0.02). In contrast, no significant associations were observed in case of PFOS for Hib (*r* = − 0.05, *p* = 0.66), tetanus IgG1 (*r* = − 0.07, *p* = 0.52) and diphtheria antibodies (*r* = − 0.02, *p* = 0.84). A further adjustment (Spearman partial correlation) for the equivalent duration of exclusive breastfeeding revealed no relevant influence of this parameter. There were no significant correlations of levels of PFHxS and PFNA with levels of the vaccine antibodies (data not shown).

**Fig. 3:**
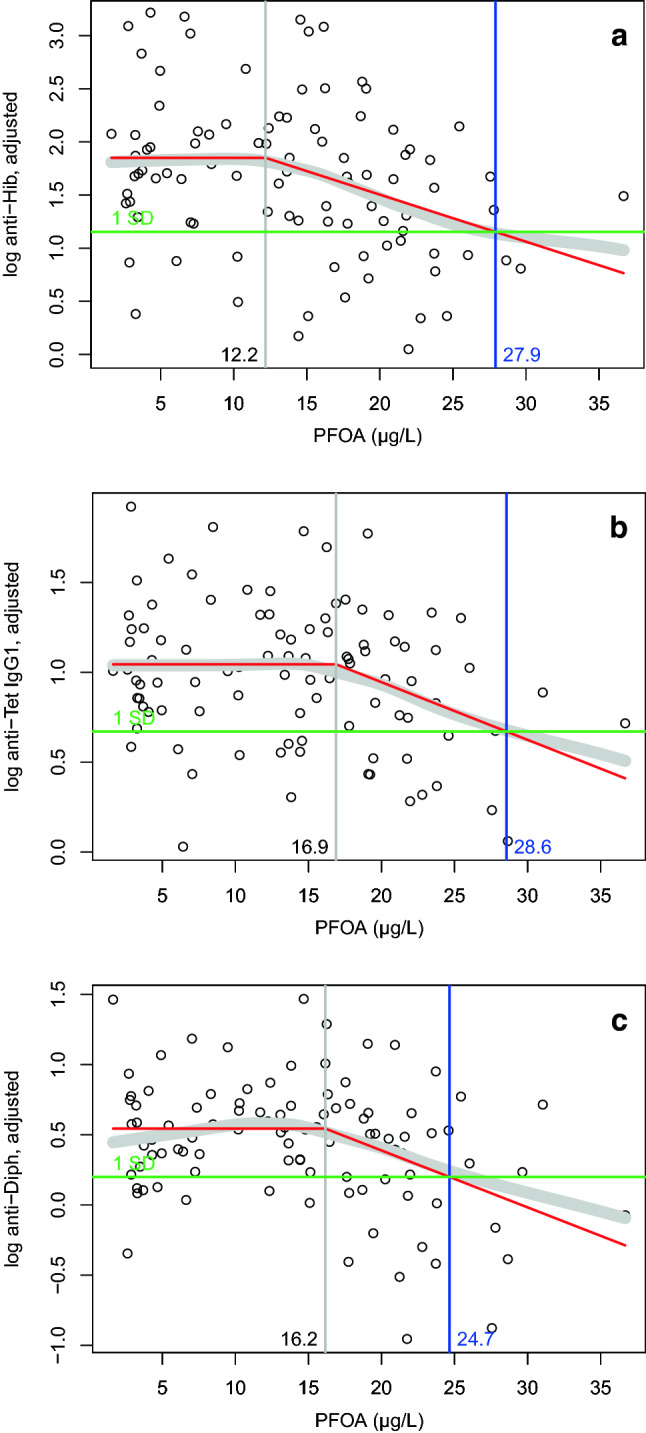
**a**–**c** Scatter plot of levels of vaccine antibodies adjusted for the number of vaccinations (in case of tetanus only) and for the time since the last vaccination for Hib (**a***n* = 98), tetanus IgG1 (**b***n* = 100) and diphtheria (**c***n* = 100), in relation to the levels of PFOA. Broad gray band: moving average. Red line: Fitted ‘knee’ function. Horizontal green line: mean minus one standard deviation of the antibody levels below the PFOA ‘knee’ level. Vertical gray line: PFOA level of the ‘knee’. Vertical blue line: PFOA level of the ‘knee’ function with antibody levels averagely lowered by one standard deviation

To quantify the PFOA concentration-dependent lowering of adjusted antibody levels, individual data are displayed for the three antibodies in Fig. 3a–c, together with the moving average revealing a ‘decrease’ roughly above concentrations of 15 µg/L. As these results support an interpretation of an effect occurring above a certain PFOA level, different approaches were used to estimate a NOAEC and the effect size above this level. Fitting a function consisting of two linear segments, a horizontal segment and—after a sharp bend—a decreasing segment (‘knee’ function, see “Methods”), revealed ‘knee’ levels (corresponding to NOEACs) for the different vaccinations between 12.2 and 16.9 µg/L, and PFOA levels between 24.7 and 29.7 µg/L corresponding to an average ‘decrease’ of antibody levels by one standard deviation of the values of children with PFOA levels below the ‘knee’ levels (see Fig. 3 and Table 3).

As can be seen especially from the individual data for antibodies against diphtheria in Fig. 3c, a higher proportion of the adjusted levels (i.e. regressed values at the time of vaccination) was below a certain level at higher PFOA levels: of the 11 children below a limit of 1 IU/mL (0 on the log-scale), just one child had a PFOA level below the ‘knee’ at 16.2 µg/L, whereas 10 children had higher PFOA levels.

To allow viewing on the data from a different perspective, quintiles and deciles for the three levels of adjusted antibodies in relation to the PFOA and PFOS levels are displayed as boxplots in supplemental Figure S1. To illustrate normal distribution within the PFOA quintiles, cumulative distribution curves are presented in supplemental Figure S2. NOAECs (between 18.9 and 22.4 µg/L) were also derived as PFOA mean of the quintiles/deciles below the one with significant difference from the first quintile/decile (*t*-test, Table 3). Testing was only done in case of a significant ANOVA, i.e. for PFOA and all three vaccine antibodies (for details see supplemental Table S2); in case of PFOS, ANOVA analyses did not reveal significant differences for the quintile/decile distributions. Comparing the means of adjusted antibody levels (i.e. the regressed values at the time of vaccination after back transformation to the linear scale) for the PFOA quintiles Q1 and Q5, a reduction on the linear scale by 86, 54 and 53% was observed in case of Hib, tetanus and diphtheria, respectively.

Regarding the other contaminants measured (Table 2) possibly acting as important confounders, correlation of antibody levels (adjusted for the time since last vaccination and the number of vaccinations in case of tetanus) with the contaminant levels revealed significant associations for I-TEq (*r* = − 0.22, *p* = 0.03) and ndl-PCBs (*r* = − 0.23, *p* = 0.02) in case of Hib only, likely resulting from the high correlation of these contaminant groups with PFOA (see above and Table S1); a multivariate analysis using the function ‘stepAIC’ in R with inclusion of I-TEq and ndl-PCBs in addition to PFOA and PFOS revealed a (highly significant) influence of PFOA only. No significant associations were observed between other contaminants and other antibody levels.

### Immune parameters directly related to the vaccine response

Besides antibody production, also the production of IFNɣ and the proliferation of ex-vivo lymphocytes after stimulation with the toxoids were available for the evaluation of the immune response to tetanus and diphtheria vaccination. These three independent biological parameters revealed significant correlations between each other (at least *r* = 0.28) for tetanus and diphtheria, respectively, with highest correlation coefficients for production of IFNɣ and proliferation (*r* = 0.65 for tetanus and *r* = 0.64 for diphtheria), both determined in the same cell culture. Levels of PFOA significantly correlated with the production of IFNɣ of ex-vivo lymphocytes after stimulation with tetanus (*r* = − 0.33, *p* = 0.01) and diphtheria (*r* = − 0.24, *p* = 0.08) toxoid (*n* = 55 only, see also Table 3). For the PFOS levels, associations were missing (*r* = − 0.10 for tetanus) and or weaker (*r* = − 0.21 for diphtheria). Comparing the means of IFNɣ levels for the PFOA tertiles Q1 and Q3 (*n* = 18 each), a reduction by 64 and 59% was observed in case of tetanus and diphtheria, respectively. No relevant associations were observed between PFOA/PFOS and lymphocyte proliferation after specific and unspecific stimulation (Table 4).

**Table 4:**
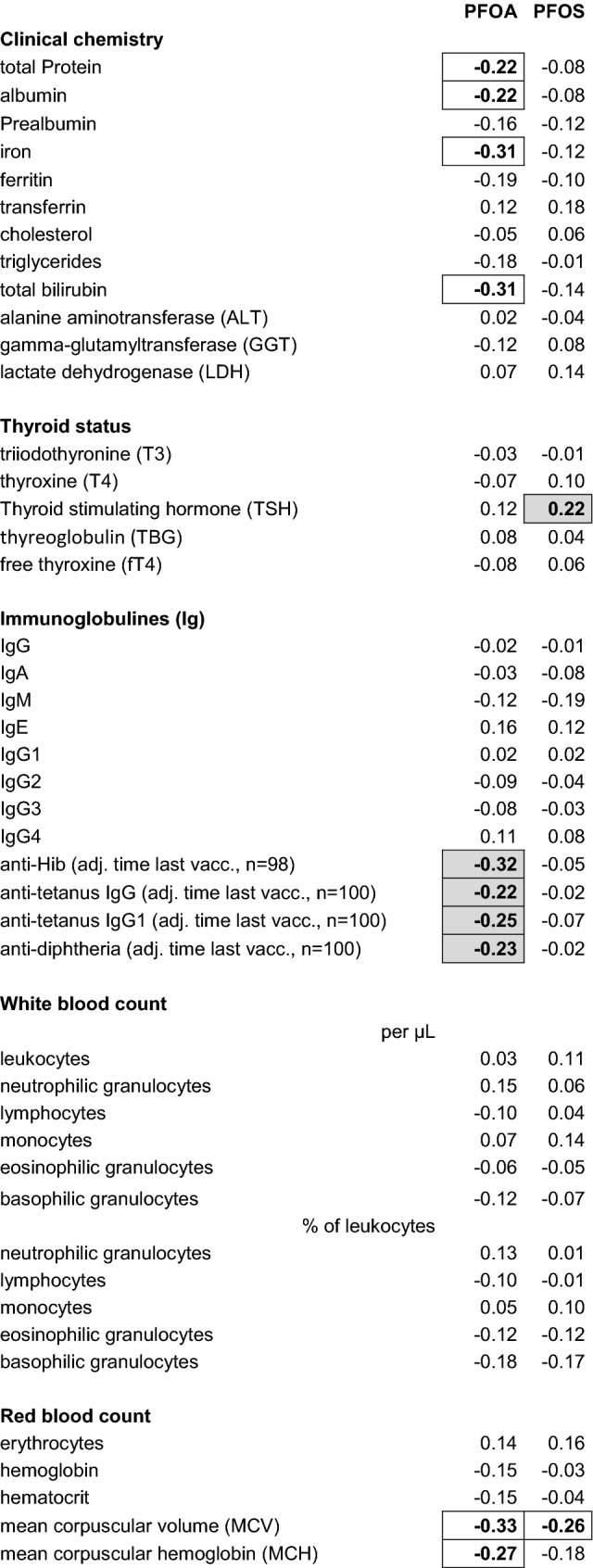

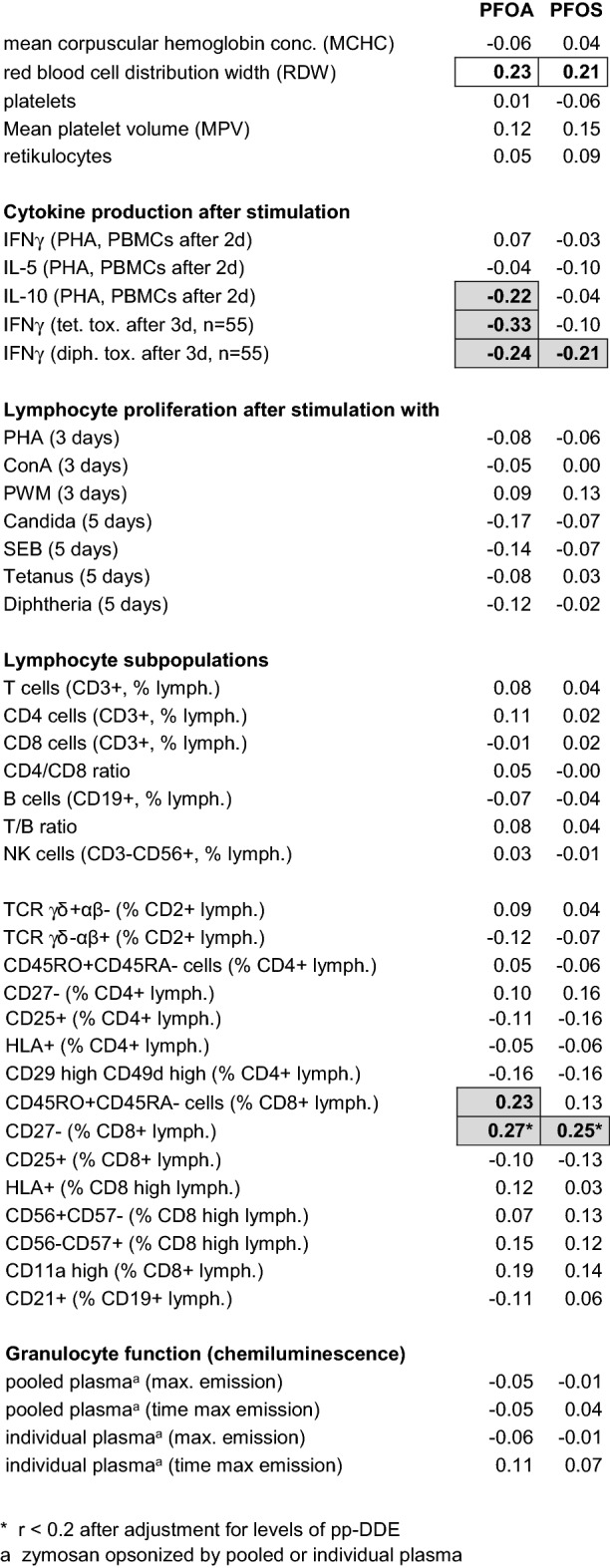
Correlation of the levels of PFOA and PFOS with the biological parameters measured (Spearman correlation coefficients)

Values above *r* = 0.2 (roughly corresponding to *p* < 0.05 in case of *n* = 101) are in bold and framed. High correlations of some nutrition-depending parameters not marked by a shadow are likely due to the high correlation of especially PFOA with the equivalent duration of exclusive breastfeeding (no significant correlation anymore after adjustment for this item). Sample size is *n* = 101 unless stated otherwise

### Other immune parameters

Correlation coefficients for the various biological parameters measured are compiled in Table 4. No significant influence was observed for the main groups of immunoglobulins (IgG, IgA, IgM, IgE) and IgG subclasses. The same holds true for white blood cell count, lymphocyte main populations (T cells, CD4/CD8 cells, B cells, NK cells), ex-vivo lymphocyte proliferation after unspecific stimulation, and granulocyte function.

Regarding the cytokine production of ex-vivo lymphocytes after unspecific stimulation with PHA, a significantly inverse association with PFOA was found for IL-10 (but not for IL-5 and IFNγ, Table 4). However, this parameter was found to have a low biological reproducibility (see above). Of the lymphocyte subpopulations investigated, significantly positive correlations with levels of PFOA were found for CD45RO^+^CD45RA^−^ cells (*r* = 0.23) and for CD27^−^ cells as percentage of CD8^+^ T cells (*r* = 0.27), however, for the latter, the correlation coefficient was below 0.2 after adjustment for levels of pp-DDE. Both surface markers were found to correlate between each other (*r* = 0.34).

### Infections and PFOA/PFOS levels

The correlations of anamnestic items regarding infections in the first year of life with PFOA and PFOS are compiled in supplemental Table S3, adjusted for the number of siblings and the equivalent duration of breastfeeding. The evaluation revealed no influence of PFOA and PFOS on infections during the first year of life. Furthermore, no significant associations were observed between levels of PFOA/PFOS and the occurrence of atopic skin diseases (data not shown) as well as the levels of CRP (supplemental Table S3).

### Other biological parameters: clinical chemistry, red blood count, thyroid status

Three nutrition-depending parameters of clinical chemistry (total protein, albumin, iron) were found to be negatively associated with PFOA, but not with PFOS (Table 4). This is likely due to the high correlation of PFOA with the duration of breastfeeding which is associated with lower protein and iron intake. The lower iron intake by breastfeeding can lead to an iron deficiency, measurable as hypochromic, microcytic anemia with higher red blood cell distribution width (RDW). Indeed, these parameters were also negatively (MCH, MCV) or positively (RDW) associated with PFOA (and in part with PFOS). An adjustment of these correlations for the equivalent duration of exclusive breastfeeding revealed disappearance of the significant associations in all the cases described. The same holds true for the inverse association between bilirubin and PFOA (data not shown).

Regarding other parameters under discussion to be effected by PFOA and/or PFOS, no significant associations were found for cholesterol, liver enzymes or thyroid parameters, apart from a significantly positive association of PFOS and the thyroid stimulating hormone (TSH, Table 4). Pathologically high cholesterol levels of more than 200 mg/dL were found in six children (5.9%, range 208–309 mg/dL) and are likely to be genetically determined; no associations of these values with PFOA/PFOS levels were observed. An exclusion of these children did not change the missing correlation of cholesterol and PFOA/PFOS. Values of cholesterol (mean ± SD) for PFOA quintiles Q1 and Q5 were 151 ± 19 and 153 ± 39 mg/dL, respectively.

## Discussion

This study has investigated relations between a broad panel of biological parameters and the relatively high internal exposure to POPs at the end of the first year of life in children breastfed for a long duration. Whereas no impact on the biological parameters measured was suggested for dioxins, PCBs and some ‘old’ pesticides (Abraham 2000), the additional measurement of PFASs and the subsequent targeted re-evaluation revealed consistent inverse associations between levels of all three vaccine antibodies investigated and levels of PFOA, in line with results in older children (Grandjean et al. 2012, 2017a, b; Granum et al. 2013). The significance of our results is unambiguous especially due to the high stability of the associations found using different methods of evaluation and due to considering the broad spectrum of other contaminants also measured in this study as possible confounders. In addition, the IFNɣ production of ex-vivo lymphocytes after stimulation with tetanus/diphtheria toxoid was also found to be negatively associated with PFOA levels. This finding indicates a more profound negative impact of PFOA on the immune system involving not only antibody-mediated but also cell-mediated immunity. Overall, the study results contribute to the cumulative evidence of a causally related effect of PFASs in humans at relatively low internal exposures.

### Relevance of a diminished immune (vaccine) response

Our evaluation indicates—in association with the levels of PFOA—a lower production of the three vaccine antibodies directly after the vaccinations. Comparing the means of levels of adjusted antibodies for PFOA quintiles Q1 vs. Q5, a considerable reduction of the adjusted antibody levels reflecting regressed values at the time of vaccination (decrease by 53 to 86%) was observed. On the other hand, one could argue that a 50% lower antibody level is already reached after one half-life which is several weeks on average in the 1-year old children of our study after two to three vaccinations against Hib, tetanus or diphtheria. The lower vaccine response is expected to result in earlier achieving of antibody levels considered not being protective anymore. Such levels, however, are difficult to define. For tetanus, according to the World Health Organization (WHO), “the minimum amount of circulating antitoxin that in most cases ensures immunity to tetanus is assay-specific. Within in vivo neutralization tests, modified ELISAs or bead-based immunofluorescence assays, concentrations at or exceeding 0.01 IU/mL are usually considered protective against disease, whereas antitoxin concentrations of at least 0.1–0.2 IU/mL are defined as positive when ELISA techniques are used for the assessment” (WHO 2018). The latter measurements are considered to be only a correlate to the surrogate of protection which is measurement of toxin-neutralizing activity in serum (WHO 2018).

At least on the population level, a diminished production of vaccine antibodies due to an environmental contaminant should in principal be considered with concern—not only with respect to the antibody levels but to the underlying changes of the cellular immune system possibly having an impact also on other important functions. Whereas the humoral immune response can easily be measured in serum/plasma samples, the extent of the involvement of the complex cell-mediated immune response is not easy to determine but more important: it is the functional basis of the immune system determining the defense against infectious agents, of which the humoral response is only a part (Siegrist 2017). The laboratory investigation of the cell-mediated immune response requires ex-vivo white blood cells which have to be processed shortly after withdrawal. In our study, a broad spectrum of parameters was investigated, and regarding a functional evaluation, the proliferation and cytokine production of lymphocytes after stimulation was of special importance. It revealed—for the first time in human ex-vivo cells—an association of PFOA level with a lower production of IFNɣ after stimulation with tetanus and diphtheria toxoid, and thereby the involvement of the cell-mediated immune system likely to finally cause the diminished production of vaccine antibody by plasma cells.

In our study, no significant/relevant associations with PFOA levels were observed for other components of the immune system (main fractions of immunoglobulins, IgG subclasses, white blood cell count, lymphocyte main populations, ex-vivo lymphocyte proliferation after unspecific stimulation, granulocyte function), apart from a positive correlation of PFOA levels and CD45RO^+^CD45RA^−^ cells as well as CD27^−^ cells as percentage of CD8^+^ T cells (but not of CD4^+^ T cells). The former population of CD8^+^ T cells comprises both T central memory (T_CM_) and T effector memory (T_EM_) cells (Sallusto et al. 2004), whereas CD27 is a known T cell costimulatory molecule (Maecker et al. 2012) and was reported to support antigen-specific proliferation (Hendriks et al. 2000). The latter may point to a diminished antigen-specific T cell activation; however, the association with levels of PFOA was not significant after adjustment for levels of pp-DDE, and enlightening conclusions can’t be drawn from these results at the moment.

### Higher risk/severity of infections due to PFAS exposure?

As a consequence of a diminished immune response to vaccines, the question of its clinical relevance in terms of an increased risk or severity of infectious diseases arises, provided that the effect of PFASs is causal and the dose is high enough. In our study, no associations were observed between levels of PFOA/PFOS measured at the age of 1 year (postnatal exposure) and the number of infections within the first year of life. This may be due to levels of PFASs being not high enough to cause this effect or due to a protective influence of the long duration of breastfeeding in the higher exposed children, if indeed an impact on the occurrence of infections is to be expected at higher levels of exposure. The few previous studies on the susceptibility to infections in young children focused on the prenatal PFAS exposure only (based on levels of PFASs in maternal blood at delivery or cord blood) and in part reported positive associations, e.g. with the prevalence of fever at the age of 1 to 4 years (Dalsager et al. 2016), while others did not find such associations (e.g. Okada et al. 2012). In this context, the window of exposure considered may be relevant, but in any case the internal dose level of PFASs. It is currently unknown, whether the production of vaccine antibodies and possibly other immune functions are further impaired in case of exposures higher than those in the studies currently available, or whether the maximal effect is only moderate not leading to a higher risk of severe diseases. Considering a high exposure to PFOA, the contamination of drinking water in West Virginia/USA, which led to a mean internal PFOA exposure in the high two-digit µg/L range (C8 cohort, Frisbee et al. 2009) did not reveal evidence of any positive association between PFOA levels during pregnancy and increasing risk for any of the categories of infection reported by the 878 mothers asked about infections in their children in the previous 12 months (C8 Science Panel 2012). Furthermore, it should be noted that in a phase 1 dose-escalation trial assessing the chemotherapeutic potential of ammonium perfluorooctanoate in 49 adult cancer patients (weekly doses of 50 to 1200 mg for 6 weeks, Convertino et al. 2018), obviously no breakdown of the immune system occurred despite plasma PFOA level in part above 1000 µM (approximately 420,000 µg/L).

### Immunotoxic potency of PFOA and PFOS in children

Internal plasma/serum levels of PFASs resulting from the general environmental contamination always occur as a mixture of different PFASs. During the last decades, PFOS und PFOA were the lead compounds, followed by PFHxS and PFNA with much lower concentrations making a relevant contribution of latter compounds to a (possible) effect of PFASs unlikely. In contrast to other studies investigating associations between levels of PFASs and vaccine antibodies in children with an internal exposure of PFOS on average (much) higher than that of PFOA, both compounds were in the same range in the 1-year-old breastfed children of our study with mean values between 15 to 17 µg/L (due to the higher accumulation of PFOA during the breastfeeding period), allowing a simple comparison of their immunotoxic potencies. While associations between levels of PFOA and levels of vaccine antibodies as well as IFNɣ production after specific stimulation were consistently related to each other, no relevant associations were observed with levels of PFOS suggesting a higher immunotoxic potency of PFOA in humans.

At first view, this seems surprising especially in consideration of results of the studies from the Faroe Islands (Grandjean et al. 2012, 2017a) suggesting an effect of PFOS, and possibly of PFOA, on the antibody response following vaccination in young children (EFSA 2018). However, this conclusion mainly results from the pattern of the exposure with PFOS levels much higher than PFOA levels (geometric means: 16.7 vs. 4.1, respectively, in the 5-year-old children of Faroe Island cohort 3, Grandjean et al. 2012), and a higher immunotoxic potency of PFOA may be masked by its high correlation with PFOS. For many of the associations between levels of PFASs at different ages and levels of vaccine antibodies, only PFOA revealed significance but not PFOS, for example in Faroe Islands cohort 5 with a median exposure level of 4.7 and 2.2 µg/L for PFOS and PFOA, respectively, at the age of 5 years (Grandjean et al. 2017a). Furthermore, benchmark dose calculations with data of Faroe Islands cohort 3 revealed much lower values for PFOA than for PFOS (Grandjean and Budtz- Jørgensen 2013).

Our results in the 1-year-old children revealed NOAECs for PFOA—depending on the method of derivation—in the range between 12 and 23 µg/L, with high consistency for the three different antibodies. However, a NOAEC of PFOA above 12 µg/L make it difficult to understand associations in other studies with mean PFOA levels much lower than 12 µg/L (e.g. Grandjean et al. 2012, 2017a). No NOAECs were derived by the authors, and the effect size was quantified as percentage of decrease in antibody levels resulting from a doubling of PFOA/PFOS levels, whereas we quantified it as percentage of decrease in antibody levels depending on the PFOA level above the NOAEC derived.

Overall, epidemiological data in young children currently available (this study and Grandjean et al. 2012, 2017a, b; Granum et al. 2013) revealed inverse associations between levels of PFOA/PFOS and levels of vaccine antibodies, but consistency regarding the immunotoxic potencies of PFOA and PFOS, the time course of diminished antibody levels as well as the dependency on the infectious agent (against the vaccination was carried) is limited for the different study groups and cohorts. This may in part be a result of different child ages and different windows of exposure for different PFOA/PFOS pattern as well as unidentified confounders. Further epidemiological studies are necessary to increase the understanding.

### Possible non-immune effects of PFOA and PFOS

In general, several biological changes are under discussion to be influenced by the background exposure to PFASs, with the most consistent finding of moderately higher levels of serum/plasma cholesterol in adults in relation to their PFOA/PFOS levels (EFSA 2018). In this context, a study involving more than 46,000 residents exposed primarily to PFOA via contaminated drinking water revealed cholesterol levels to be higher by approximately 10 mg/dL, comparing the lowest PFOA/PFOS levels and PFOA/PFOS levels of about 50 µg/L (Steenland et al. 2009). Despite the internal exposure to PFOA and PFOS of the children in our study was within this range, cholesterol levels were unchanged in relation to the levels of PFOA and PFOS. This may reflect age-dependent susceptibility. To our knowledge, no other data are available for this issue in infants and toddlers.

Regarding a possible impact of PFASs on thyroid parameters, a weak positive association between PFOS and TSH was observed. Study results of others indicate some evidence for an effect of PFASs on thyroid parameters, however, no clear patterns emerge (Rappazzo et al. 2017).

## Conclusions

The present study has many strengths with respect to the explicit in- and exclusion criteria for healthy children in a small age window at the end of the first year of life which is not only relevant regarding the relatively high levels of contaminants in children breastfed for a long duration, but also relevant regarding the vaccination schedule and the possibly higher susceptibility of the developing immune system. A broad panel of (immune) parameters was investigated, thereby applying high standards regarding the quality of measurements as well as the stability and biological reproducibility over time. Furthermore, the relatively high background levels at the end of the 1990s (as compared to the current levels), with about the same mean levels of PFOA and PFOS, improved the meaningfulness regarding possible toxicological effects. In addition, relevant other persistent organic contaminants including dioxins were also measured and considered as possible confounders. Last but not least, this is a targeted evaluation with the focus on associations of levels of PFASs with biological parameters observed in other studies.

We confirmed results observed in other studies for the associations of levels of PFOA (but not of PFOS) with levels of vaccine antibodies. This was found with high consistency for all three infectious agents against which the vaccinations were carried out, including comparable values for the NOAECs derived and the effect sizes. Furthermore, the overall plausibility is supported by the findings of a reduced IFNɣ production of ex-vivo lymphocytes after specific stimulation which points to a primary involvement of the cell-mediated immune system. Despite causality cannot be proven in cross-sectional studies, our results contribute to the cumulative evidence of a causally related effect of PFASs at least in (young) children and infants who may have a higher vulnerability, as their immune system is developing. In view of the broad spectrum of biological parameter investigated in our study, the immune system seems to be the most susceptible system for (possible) effects of PFASs in 1-year-old children.

Such effects would lead to a shorter duration of antibody levels considered as protective. This may be without consequences for the effectiveness of vaccinations, as safety margins may be high enough and may tolerate a decreased production of vaccine antibodies with the extent observed, if the vaccination schedule is followed. Nevertheless, any negative impact on the immune system has to be considered principally as undesirable, as the relevance in terms of clinical categories (e.g. higher rate of unprotected subjects in the population, higher rates or higher severity of infections in general) generally is difficult to prove.

In view of the NOAECs derived for PFOA in this study (lowest value: 12.2 µg/L as ‘knee’ level for Hib antibodies), a considerable proportion of the breastfed children (79%) was above this value in our study at the end of the 1990s in Germany. Data on time trends (e.g. Vestergren and Cousins 2009; Yeung et al. 2013) revealed this period to be likely the one with the highest internal exposure to PFOA. Due to management measures, the exposure in Germany is distinctly lower since then (PFOA in study mothers who did not breastfed: mean 4.9 µg/L; PFOA in Munich 2016: mean 1.2 µg/L, maximum 3.7 µg/L in 158 adults, Fromme et al. 2017). Therefore, children in Germany nowadays breastfed for a long duration are for the most part not expected to reach PFOA levels of 12 µg/L or higher at the end of the breastfeeding period. This applies for the current environmental background exposure, but not for hotspot regions with additional local contamination. Depending on the extent of the internal exposure in these regions, effects of PFOA and other PFASs possibly have to be weighed against the wide-ranging benefits of breastfeeding (Victora et al. 2016). In this context, no scientific body in the world has so far recommended restricted breastfeeding.

Finally, since most studies in this field are cross-sectional, data need to be interpreted with caution. More insight is needed into possible mechanisms of action, dose–response relationships and the clinical relevance. Therefore, further intensive laboratory and epidemiological research (including prospective studies) is necessary to improve the understanding.

## Electronic supplementary material

Below is the link to the electronic supplementary material.

Supplementary file1 (PDF 790 kb)

## Data Availability

In accordance with data protection regulations, access restrictions apply to the raw data underlying the findings of the present study, and thus they cannot be made publicly available.
