# Supplementary material for: Internal exposure to perfluoroalkyl substances (PFASs) and biological markers in 101 healthy 1-year-old children: associations between levels of perfluorooctanoic acid (PFOA) and vaccine response
Source: Arch Toxicol. 2020 Mar 29;94(6):2131–47. doi: 10.1007/s00204-020-02715-4 (PMC7303054; doi:10.1007/s00204-020-02715-4)

Table S1

Spearman`s rank correlation coefficients for the correlation of plasma/blood levels the persistent contaminates between each other.

	**PFOA**	**PFOS**	**PFHxS**	**PFNA**	**I-TEq**	**PCBs**	**ß-HCH**	**pp-DDE**	**Hg**	**Cd**
**PFOS**	0.67									
**PFHxS**	0.51	0.42								
**PFNA**	0.72	0.86	0.46							
**I-TEq**	0.67	0.46	0.24	0.54						
**PCBs**	0.72	0.49	0.43	0.61	0.78					
**ß-HCH**	0.61	0.46	0.24	0.54	0.80	0.78				
**pp-DDE**	0.57	0.41	0.06	0.47	0.79	0.64	0.77			
**Hg**	0.08	0.00	-0.01	0.08	0.11	0.14	0.10	0.12		
**Cd**	0.02	-0.10	0.12	-0.04	-0.01	0.09	-0.03	-0.03	0.17	
**Pb**	0.23	0.05	0.09	0.12	0.26	0.20	0.23	0.20	0.00	0.08

Table S2 No observed adverse effect concentration (NOAEC) for PFOA derived as mean of the highest-dose quantile (yellow) below the first one showing a significant difference to the lowest-dose quantile.

anti-Hib

Quintiles

Anova p 0.001

	group mean	N	mean	SD	p value
Q1	3.4	20	1.84	0.68	
Q2	8.5	20	1.84	0.71	0.98
Q3	14.8	20	1.84	0.84	0.98
Q4	19.4	20	1.50	0.55	0.09
Q5	25.7	18	1.19	0.60	0.003

Deciles

Anova p 0.002

	group mean	N	mean	SD	p value
Q1	2.8	10	1.78	0.58	
Q2	4.0	10	1.90	0.79	0.70
Q3	6.6	10	1.97	0.78	0.56
Q4	10.4	10	1.71	0.64	0.80
Q5	13.7	10	1.77	0.78	0.96
Q6	15.9	10	1.91	0.92	0.72
Q7	18.3	10	1.68	0.66	0.73
Q8	20.5	10	1.33	0.38	0.06
Q9	23.0	10	1.10	0.70	0.03
Q10	29.1	8	1.30	0.46	0.07

anti-Tetanus (IgG1)

Anova p 0.01

	group mean	N	mean	SD	p value
Q1	3.4	20	1.07	0.31	
Q2	8.5	20	1.04	0.44	0.77
Q3	14.5	20	1.02	0.39	0.67
Q4	18.9	20	1.02	0.35	0.61
Q5	25.3	20	0.74	0.36	0.003

Anova p 0.013

	group mean	N	mean	SD	p value
Q1	2.8	10	1.14	0.39	
Q2	4.0	10	1.00	0.20	0.34
Q3	6.6	10	0.89	0.49	0.22
Q4	10.4	10	1.19	0.35	0.79
Q5	13.6	10	0.87	0.37	0.13
Q6	15.5	10	1.18	0.36	0.83
Q7	17.8	10	1.13	0.21	0.93
Q8	20.0	10	0.91	0.42	0.22
Q9	22.4	10	0.81	0.35	0.07
Q10	28.1	10	0.66	0.37	0.01

anti-Diphtheria

Anova p 0.004

	group mean	N	mean	SD	p value
Q1	3.4	20	0.50	0.41	
Q2	8.5	20	0.57	0.29	0.56
Q3	14.5	20	0.64	0.36	0.26
Q4	18.9	20	0.40	0.45	0.49
Q5	25.3	20	0.13	0.53	0.02

Anova p 0.006

	group mean	N	mean	SD	p value
Q1	2.8	10	0.58	0.49	
Q2	4.0	10	0.42	0.32	0.40
Q3	6.6	10	0.47	0.31	0.57
Q4	10.4	10	0.66	0.26	0.65
Q5	13.6	10	0.57	0.24	0.95
Q6	15.5	10	0.71	0.45	0.54
Q7	17.8	10	0.38	0.38	0.33
Q8	20.0	10	0.43	0.52	0.52
Q9	22.4	10	0.16	0.57	0.10
Q10	28.1	10	0.11	0.51	0.05

Table S3

Correlation of the levels of PFOA and PFOS with anamnestic items regarding infections in the first year of life as well as CRP, adjusted for the number of siblings and the equivalent duration of breastfeeding (Spearman partial correlation coefficients; please note that the cohort size of n=101 implies that a p=0.05 roughly corresponds to r=0.2).

	**PFOA**	**PFOS**
Month of first infection	-0.18	0.01
Total number of infections	0.01	0.13
Number infections with fever	-0.10	0.09
Number antibiotic treatments	-0.08	0.06
Antibiotic ever	-0.09	0.02
Number Otitis media	0.01	0.10
Otitis media ever	0.01	0.08
3-day-fever already	-0.05	-0.04
Number pneumonia	-0.08	-0.01
Pneumonia ever	-0.08	-0.01
Number diarrhoea	0.02	-0.17
Diarrhoea ever	0.02	-0.18
Varicella already	-0.02	-0.16
Napkin candidiasis ever	0.06	0.10
Oral candidiasis ever	0.09	0.09
CRP	0.02	0.08

Figure S1

Boxplots of adjusted antibody levels in relation to levels of PFOA (this page) and PFOS (next page) as quintiles and deciles for Hib, tetanus (IgG1) and diphtheria.

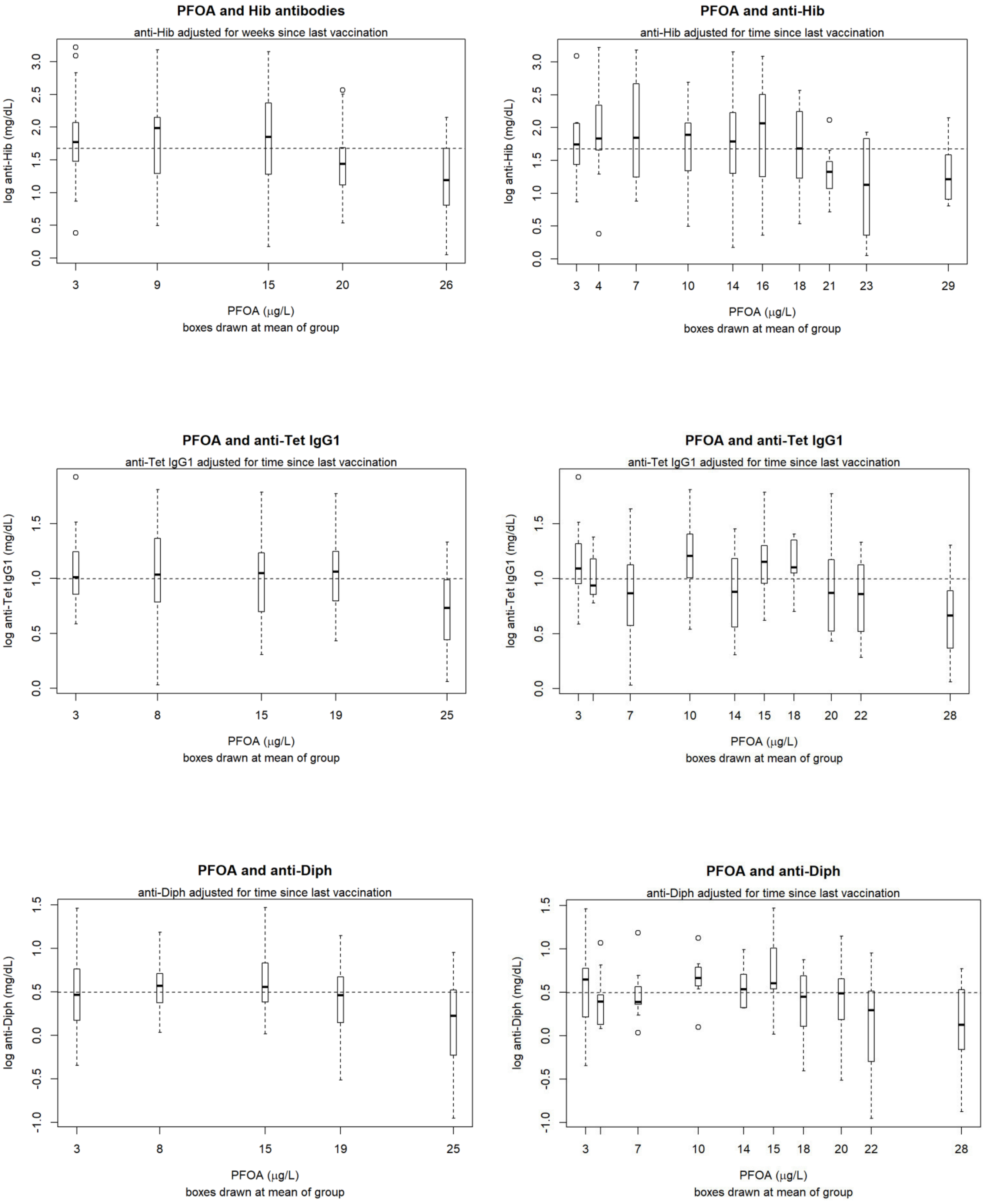

Figure S1 (continued)

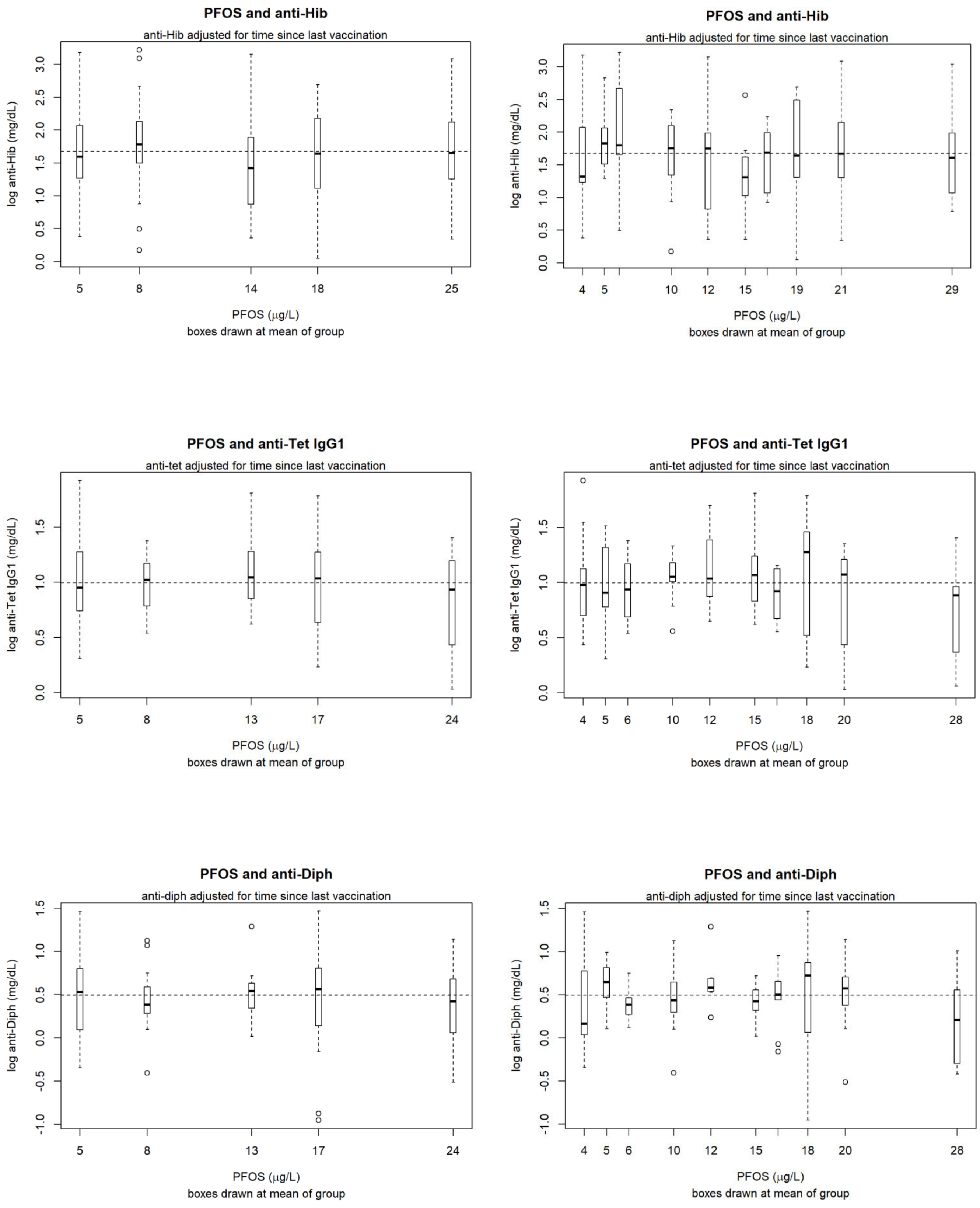

Figure S2 Empirical cumulative distribution functions of all PFOA quintiles together with the respective fitted normal distribution curves

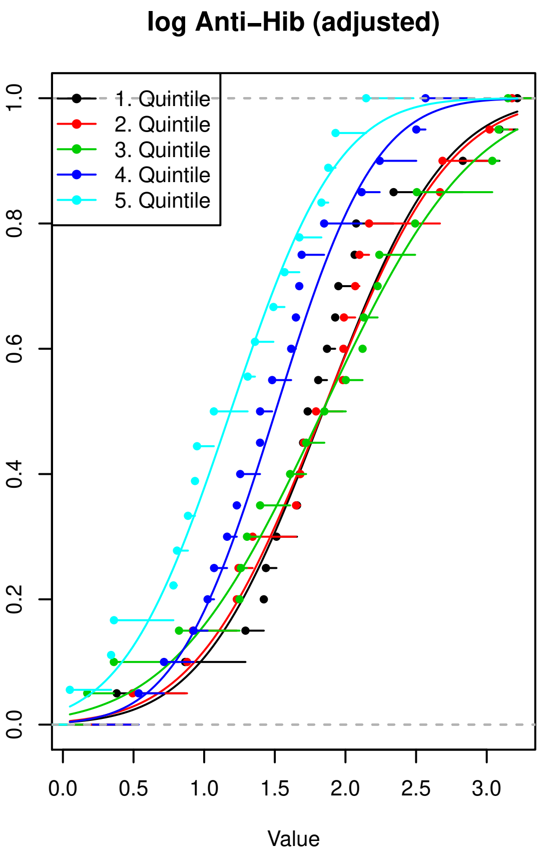

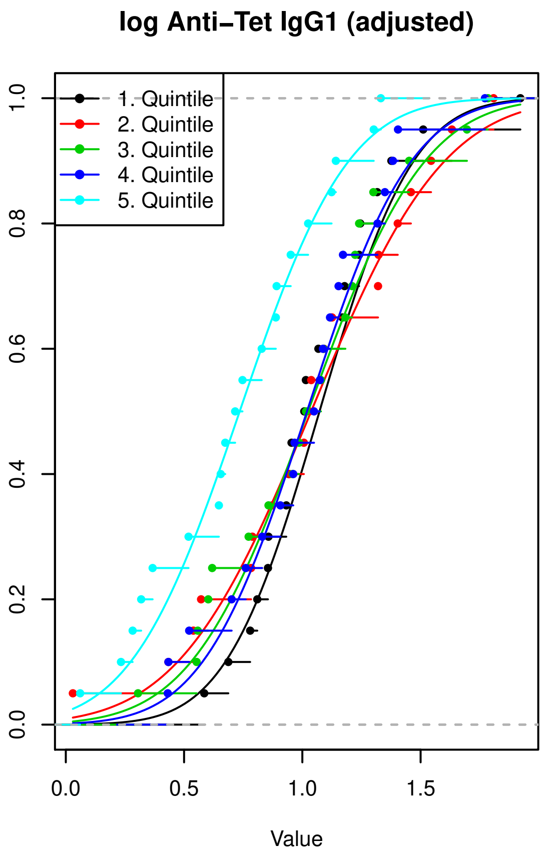

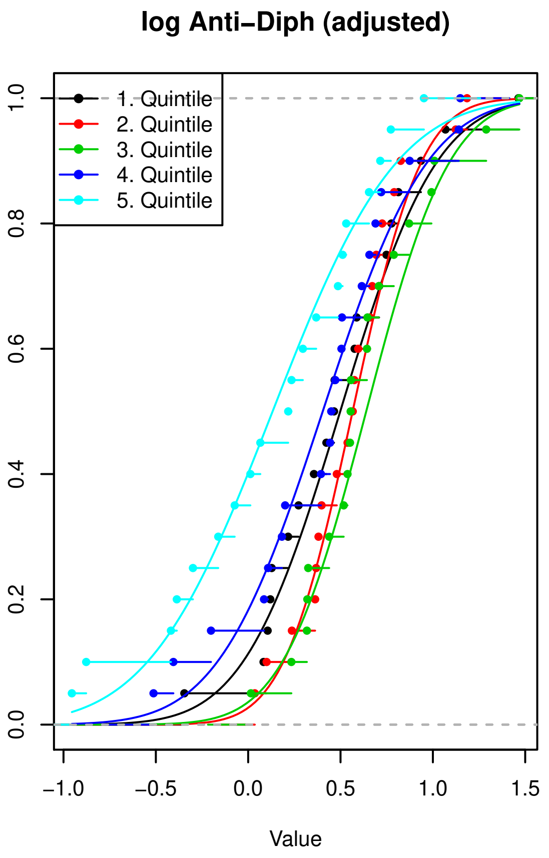

Supplement: Supplementary file 1 — Supplementary file1 (PDF 790 kb) [file 204_2020_2715_MOESM1_ESM.pdf]
